# High-molecular-weight polymers for protein crystallization: poly-γ-glutamic acid-based precipitants

**DOI:** 10.1107/S0907444908021616

**Published:** 2008-08-13

**Authors:** Ting-Chou Hu, Justyna Korczyńska, David K. Smith, Andrzej Marek Brzozowski

**Affiliations:** a Department of Chemistry, University of York, York YO10 5YW, England; b Structural Biology Laboratory, University of York, York YO10 5YW, England

**Keywords:** protein crystallization, crystallization precipitants, crystallization screens, high throughput

## Abstract

Protein crystallization has been revolutionized by the intro­duction of high-throughput technologies, which have led to a speeding up of the process while simultaneously reducing the amount of protein sample necessary. Nonetheless, the chemistry dimension of protein crystallization has remained relatively undeveloped. Most crystallization screens are based on the same set of precipitants. To address this shortcoming, the development of new protein precipitants based on poly-γ-­glutamic acid (PGA) polymers with different molecular-weight ranges is reported here: PGA-LM (low molecular weight) of ∼400 kDa and PGA-HM (high molecular weight) of >1000 kDa. It is also demonstrated that protein precipitants can be expanded further to polymers with much higher molecular weight than those that are currently in use. Furthermore, the modification of PGA-like polymers by covalent attachments of glucosamine substantially improved their solubility without affecting their crystallization properties. Some preliminary PGA-based screens are presented here.

## Abstract 2

High-molecular-weight poly-γ-glutamic acid-based polymers have been synthesized, tested and adopted for protein crystallization.

## 1. Introduction

The field of protein crystallization has undergone an extraordinary transformation in the last decade. High-throughput (HT) screening technologies have enabled more rapid determination of protein structures (often in a matter of hours) and reduced the consumption of biological material. Other innovations include the development and adaptation of protein-handling robots and liquid dispensers. As a result, the crystallization process can now be almost entirely automatic, while the single trial (‘condition’) volume of the protein has been reduced from the typical manual 1 µl setup to ∼50–150 nl. The efficiency of protein crystallization can be increased even further by the application of automated micro­diffusion devices (Hansen *et al.*, 2002 ▶; Chen *et al.*, 2007 ▶). Liquid-handling robots are also taking over the preparation of screen solutions. These advances have been accompanied by progress in other crystallization-related consumables, especially in the area of crystallization plates; more than 30 formats of these are currently available. Furthermore, the single precipitant volume in the sitting drop-like setup may be minimized to ∼1.2 µl, while also accelerating the crystal-growth process (Korczyńska *et al.*, 2007 ▶). Although dialysis-based setups have not been satisfactorily adapted to HT technologies, counter-diffusion capillary-based devices have already been designed and implemented (Garcia-Ruíz *et al.*, 2002 ▶; Garcia-Ruíz, 2003 ▶; Ng *et al.*, 2003 ▶). Traditional seeding techniques have also been adapted to HT formats, including both protein-derived and artificial nucleants (see, for example, Bergfors, 2003 ▶; D’Arcy *et al.*, 2007 ▶; Chayen *et al.*, 2006 ▶).

In contrast to these innovations, progress in the development of chemical tools for crystal growth has lagged behind. It is likely that there are more than 100 screens available today (a summary of the most common 92 formulations can be found at http://xray.bmc.uu.se/markh/php/xtalscreens.php?func=lookup&screen_name=Expand+List; courtesy of Mark Harris, Uppsala), but most contain similar types of salts, polymers (*e.g.* PEGs, Jeffamines) and additives, albeit at different ratios, pHs and concentrations. One notable exception is the recent formulation of the Silver Bullets screen, which utilizes a plethora of new (or previously reported) additives. These have been shown to be useful in the formation of crucial crystal contacts or enhancing protein conformations (McPherson & Cudney, 2006 ▶; Larson *et al.*, 2007 ▶).

An alternative to polymer-based precipitants are organic salts (*e.g.* sodium malonate, oxalate, formate; McPherson, 2001 ▶). Novel chemistry in protein crystallization also includes lipids in the form of lipidic cubic phases (Landau & Rosenbusch, 1996 ▶) for membrane proteins. However, these developments only underline the lack of new polymer-based precipitants. The only recently reported polymers that have had a significant impact on the efficiency of crystallization screens are the monomethyl PEGs, which appeared 14 y ago (Brzozowski & Tolley, 1994 ▶), Jeffamine M-­600, polyethyleneimine (Cudney *et al.*, 1994 ▶), polyacrylic acid, polyvinylpyrolidone, polypropylene glycol, polyvinyl alcohol, PEG dimethyl ether (Patel *et al.*, 1995 ▶) and penta­erythritol propoxylate (Gulick *et al.*, 2002 ▶).

**Figure 1:**
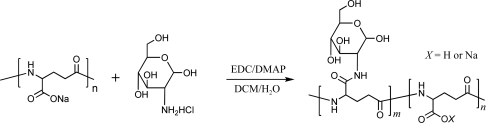
Synthesis scheme of poly-γ-glutamate–glucosamine conjugates *via* amide coupling in a two-phase system using EDC/DMAP (Ho *et al.*, 1995 ▶). The molecule on the left shows the original unmodified PGA.

In the face of this discrepancy, we embarked on a project to design, synthesize, test and implement new varieties of polymers for protein crystallization. Our primary goal was to develop new precipitants/media for the crystallization of membrane proteins. Owing to the extremely challenging character of these proteins this area suffers most from the shortage of chemical tools, although new chemical media for globular proteins would also be desirable. Here, we report the application of poly-γ-glutamic acid (PGA) based polymers (Fig. 1 ▶) to the crystallization of globular and membrane proteins. This is also the first report of the successful use of polymers with a molecular weight well over 1000 kDa in the process of protein crystallization. Because most conventional polymeric precipitants are limited to a maximum of 20 kDa molecular weight (*e.g.* PEG 20K), our results open up new possibilities for macromolecular crystallization. Although the application of high-molecular-weight polymers (∼400–700 kDa) has previously been reported for low- and high-viscosity carboxymethylcellulose (CMC; Patel *et al.*, 1995 ▶), there are no screens based on CMC as the main precipitant.

Interest in PGA-like polymers stemmed from our search for gel-forming hydrophilic macromolecules with high swelling properties. It was vital that they should also be nontoxic, nondenaturing and readily accessible for research. Trials with several different polymers [*e.g.* poly(l-lactide), poly(ether amide), poly(butylene terephthalate) and poly(hydroxy­butyrate)] indicated the PGA was promising not only for gel formation but also for protein crystallization.

PGA is a naturally occurring biopolymer synthesized from l-glutamic acid by *Bacillus subtilis* var. *natto* (Hara & Ueda, 1982 ▶). *B. subtilis* is the preferred source of this macromolecule as its chemical synthesis is not very efficient (Sanda *et al.*, 2001 ▶). PGA is a main component of natto (a Japanese health food) and its polyanionic and nontoxic properties also make it an important and widely used component in food additives, cosmetics, water treatments and natural biocides. It is commercially available in two different molecular-weight ranges: low-molecular-weight PGA (PGA-LM) of ∼200–400 kDa and high-molecular-weight PGA (PGA-HM) with an average range of ∼1000–2000 kDa. As the natural PGAs are linear structures, we have also derivatized these polymers to achieve even greater swelling properties and more branched versions of them.

Here, we describe the application of PGA and its derivatives in protein crystallization and propose a strategy for crystallization screens based on this polymer.

## 2. Material and methods

### 2.1. Preparation of the PGA for the template screen

PGA was purchased from Vedan Enterprise Corp., Taiwan. PGA-LM was poly-γ-PGA (Na^+^ salt form) cosmetic grade LM [OD_400_ maximum 0.07 for 4%(*w*/*v*) aqueous solution] with an average MW of ∼200–400 kDa, with a particle size of 100% through 100 mesh. PGA-HM was poly-γ-PGA (Na^+^ salt form) cosmetic grade HM [OD_400_ maximum 0.07 for 4%(*w*/*v*) aqueous solution] with an average MW of >1000 kDa, with a particle size of 100% through 100 mesh. PGA-LM stock solutions in deionized water were 20–50%(*w*/*v*), while PGA-HM stocks were 5–20%(*w*/*v*). All other chemicals were from Sigma–Aldrich.

### 2.2. Preparation and synthesis of PGA–glucose conjugates

**Figure 2:**
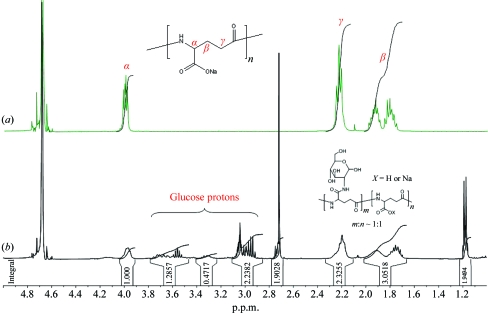
^1^H NMR spectra of poly-γ-glutamic acid (*a*) and its synthesized glucosamine conjugate (*b*). The ratio of the incorporated carbohydrate is estimated by integration of the glutamic and glucose protons. In this particular case, the ratio of the glucosamine-conjugated and non-glucosamine-conjugated glutamic segments is about 1:1.

Glucose units in the form of glucosamine were incorporated into both types of PGA *via* amide coupling (see Fig. 1 ▶). The amide-coupling reaction was carried out in a two-phase system (Ho *et al.*, 1995 ▶) using EDC [*N*-(3-dimethylaminopropyl)-*N*′-ethyl carbodiimide hydrochloride] and DAMP [4-(dimethyl­amino)pyridine]. In the case of PGA-LM, 1.51 g of the biopolymer, 1.08 g (5 mmol) glucosamine hydrochloride and 1.05 g (5 mmol) EDC were loaded into a 250 ml round-bottom flask with 100 ml deionized water. 4-(Dimethylamino)pyridine (153 mg, 1.25 mmol) and triethylamine (0.7 ml, 5 mmol) were dissolved in 50 ml dichloromethane and then added to the reaction flask. The mixture was stirred for 24 h. The aqueous phase was transferred to a separation funnel and washed with 50 ml dichloromethane. The aqueous solution was further dialysed against 0.05 *M* HCl and subsequently against de­ionized water. The final product was obtained through vacuum drying as a white solid with a 62% (1.6 g) yield. The ratio between the glutamic acid unit and conjugated glucose was estimated by ^1^H NMR (see Fig. 2 ▶): ^1^H NMR (400 MHz, D_2_O, p.p.m.), 4.18 (glutamic α proton, 1H, m), 3.8–2.9 (glucose protons, 7H, m), 2.20 (glutamic γ protons, 2H, m), 1.98–1.65 (glutamic β protons, 2H, m); ^13^C NMR (100 MHz, D_2_O, p.p.m.), 175–174 (carbonyl C atoms), 91.2 (glucose C6), 75.9, 74.1, 71.8, 69.8 (glucose C3–5), 61.8 (glucose C1), 55.5 (glucose C2), 52.1 (glutamic α carbon), 31.5 (glutamic β carbon), 26.5 (glutamic γ carbon).

### 2.3. Test proteins

Lysozyme (Sigma), *Trichoderma reesei* xylanase (Hampton Research) and *Streptomyces rubiginosus* glucose isomerase (Hampton Research) were used as test proteins. Prior to use with the PGA solutions, the crystallizability of these proteins was optimized (as described in Korczyńska *et al.*, 2007 ▶) on 24-­well Linbro tissue plates with 1 ml well solution and 1 µl protein + 1 µl precipitant solution and on 96-well Greiner CrystalQuick plates with standard drops of 150 nl protein + 150 nl precipitant and 100 µl reservoir solution. 96-well setups were performed with Mosquito (TTP Labtech, UK). The conditions for the three test proteins were as follows: lysozyme, 30 mg ml^−1^ protein in 50 m*M* sodium acetate buffer pH 5.0, 0.1 *M* sodium acetate buffer pH 4.5, 1.0 *M* NaCl; xylanase, 35 mg ml^−1^ protein in 50 m*M* Tris–HCl pH 7.5, condition *A* [0.1 *M* Tris–HCl pH 8.5, 0.2 *M* LiCl, 20%(*w*/*v*) PEG 4000]; glucose isomerase at 38 mg ml^−1^ in 50 m*M* Tris–HCl pH 7.5, condition *A* [0.1 *M* Tris–HCl pH 7.0, 0.2 *M* MgCl_2_, 20%(*v*/*v*) PEG 400], condition *B* [0.1 *M* Tris–HCl pH 7.0, 0.2 *M* MgCl_2_, 23%(*v*/*v*) MPD]. All crystallizations were carried out at room temperature (292 ± 0.5 K).

In addition to the test proteins, the PGA solutions and screens were tested on ten previously uncrystallized proteins, including two membrane proteins, from ongoing projects at the York Structural Biology Laboratory (YSBL) and the Department of Biology, University of York.

### 2.4. Preparation of the screens

Detailed compositions of the screens is provided in Tables 1–­5. All screens were made up to 90% of their final volume, allowing a further top-up of the remaining 10% volume with 1 *M* stock buffer at the desired pH, similar to the strategy used in CSS screens (Brzozowski & Walton, 2001 ▶).

## 3. Results and discussion

### 3.1. Preliminary evaluation of PGAs in protein crystallization

Initial solubility trials of the PGAs showed that the stock solutions of both biopolymers could be prepared at concentrations that were high enough for protein-crystallization trials. As expected, it was possible to make a 50%(*w*/*v*) stock of PGA-LM, but the PGA-HM stock could only be dissolved to 10–20%(*w*/*v*). However, for practical reasons the PGA-LM stocks were frequently used at 20–30%(*w*/*v*). The viscosity of PGA solutions [*e.g.* 150 mPa s for 4%(*w*/*v*) PGA-HM and 50 mPa s for 4%(*w*/*v*) PGA-LM (both in water; Vedan Technical Data); for comparison, 50%(*w*/*v*) PEG 6K has a viscosity of 100 mPa s] allowed manual dispensing. Their compatibility with large-volume liquid-handling robots employed for screen making was not tested here but should be feasible.

It has to be stressed that the three test proteins (lysozyme, glucose isomerase and xylanase) are all easily crystallizable. They were used here not to assess the increased (if any) crystallization efficiency of the PGAs but to check whether PGA-based polymers were even feasible to use, *i.e.* that they would not inhibit crystal growth. As the real usefulness of the PGAs can only be established if they work for real (not typical test) proteins, the PGAs were partially validated here on ten proteins from ongoing projects within the YSBL, including two bona fide membrane proteins.

**Figure 3:**
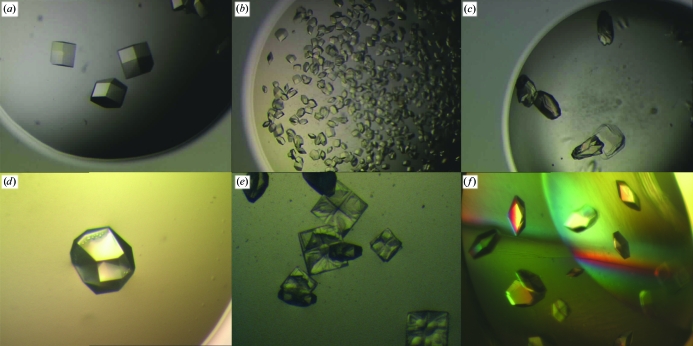
Examples of lysozyme crystal growth from PGA-LM. (*a*) 1%(*w*/*v*) PGA-LM, 0.2 *M* NaCl pH 4.5. (*b*) As (*a*) but with 5%(*w*/*v*) PGA-LM. (*c*) As (*a*) but with 10%(*w*/*v*) PGA-LM; crystals grow here as easily as in (*a*) but frequently intergrow into each other and cluster. (*d*) As (*a*) but with 1%(*w*/*v*) pectin. (*e*) As (*a*) but with 10%(*w*/*v*) pectin; a significant slowing of crystal growth can be observed with ‘step/spiral-like’ individual crystal faces. (*f*) As (*a*) but with 10%(*w*/*v*) gum arabic; some three-dimensional in-gel-like distribution of the crystal can be observed here despite the relatively low viscosity of the solution.

At first, both PGAs were used as additives [0.7–2%(*w*/*v*)] in the test conditions established for the three test proteins. As they did not visibly interfere with (or inhibit) the crystal growth of the test proteins, they were tested as the main precipitants at 1–20%(*w*/*v*) for PGA-LM and 0.5–5%(*w*/*v*) for PGA-HM over a range of pH values from 4.5 to 7.8 with and without different salts (average salt concentration: 0.2 *M*). A plethora of crystals from all test proteins were obtained over the whole range of PGA concentrations (with or without salts; Figs. 3 ▶ *a*–3*c*). As the result of this preliminary screening, the first simple template PGA-based screen was formulated (Table 1 ▶). Practically all conditions (*A*–*H*) with all salts yielded test crystals over the three selected pH values, with an obvious preference for pH 4.5 in the case of lysozyme and pH 7.8 for glucose isomerase and xylanase.

It should be emphasized here that PGAs are polyanionic polymers and have substantial chelating properties which, for example, explain their use in water treatment. Therefore, the role of any inorganic/organic salts used in conjunction with the PGAs may be quite different from that they may have in classical (*e.g.* PEG-based) screens. However, as the salts used in the template PGA screen are at high molar concentrations, they are likely to still exhibit protein-crystallization activities, whereas at millimolar levels their cations would be effectively scavenged from the solution by the PGA.

### 3.2. Carbohydrate–PGA-based crystallization screening

As the template PGA screen was very effective in crystallization of the test proteins, it was modified further to enhance its swelling properties by adding pectin and gum arabic, respectively. Both of these carbohydrates have been found to be useful additives for slowing down crystal growth and minimizing nucleation (AMB, unpublished results). Test crystals were obtained over a wide range of pectin/gum arabic concentrations (Figs. 3 ▶ *d*–3*f*), but the best results (crystals with well shaped morphology) were obtained for 1%(*w*/*v*) pectin and 5%(*w*/*v*) gum arabic. Interestingly, some of the PGA/gum arabic solutions resulted in suspension-like crystal growth in the whole volume of the drop that is more typical of gel-like sedimentation-free crystallizations (Fig. 3 ▶ *f*; see, for example, Garcia-Ruíz *et al.*, 2001 ▶; Willaert *et al.*, 2005 ▶). However, the ‘working’ viscosities of the PGA/gum arabic media used in these crystallization remained very similar to those of the PGA solutions without the carbohydrates. In some cases (Fig. 3 ▶ *e*), slowing down the crystal growth produced crystals with visible spiral/step-like growth of crystal faces, which suggests that this could also be interesting for crystal-growth studies.

The template PGA screen was modified further to incorporate the best results obtained with the addition of the carbohydrates and 1%(*w*/*v*) pectin and 5%(*w*/*v*) gum arabic were added to all conditions. The resulting carbohydrate–PGA screen was formulated and tested. Both template PGA and carbohydrate–PGA screens were set up at three different pH values: 4.5 (0.1 *M* sodium acetate), 6.5 (0.1 *M* cacodylate) and 7.8 (0.1 *M* Tris–HCl). The refined conditions are summarized in Table 2 ▶ in terms of the most effective crystallization of the test proteins. The addition of the carbohydrates in the carbohydrate–PGA screen slowed (by ∼1–3 d) crystal growth of the test proteins, as expected. However, application of the carbohydrate–PGA screen to the ongoing YSBL projects was not visibly beneficial. Therefore, pectin and gum arabic were not used further in refinement of the PGA-based screens, although their exploitation may still be helpful for some proteins. They should not be disregarded in further applications and developments, especially in the optimization of crystal growth.

### 3.3. Covalent modification of the PGAs

As the general applicability of the PGAs in the crystallization process had been established, we also explored the branching of these biopolymers, this time not by the addition of other chemical components but by covalent modification. Several glucosamine derivatives of both PGAs were synthesized using amide-coupling reactions. The resulting glucos­amine PGAs were obtained (Fig. 1 ▶) with different glutamic acid:glucosamine molar ratios for both PGA-LM and PGA-HM. Subsequently, we focused on glucosamine PGA polymers with an average 2:1 glutamic acid:glucosamine molar ratio, which was the most straightforward to control during the synthesis, purification and characterization of the resulting modified polymer. Other carbohydrates were considered for further modifications but their organic synthesis proved not to be cost-effective for large-scale crystallization trials.

As expected, the solubility of the glucosamine-modified branched PGAs increased substantially. From a solubility point of view, the conjugation with glucosamine was un­necessary in the case of PGA-LM as 20–50%(*w*/*v*) stock solutions were easily obtained. However, modification of the PGA-HM to glucosamine-PGA-HM significantly elevated its solubility and allowed the preparation of 40%(*w*/*v*) stock solutions [in comparison with 10–20%(*w*/*v*) for unmodified PGA-HM]. Although both glucosamine-PGA-LM and glucosamine-PGA-HM were also effective in the crystallization of the test proteins in ranges very similar to those achieved for the non-modified PGAs (Table 3 ▶ shows one such screen), their widespread applications were limited owing to the cost of their synthesis. Nonetheless, they were efficiently and economically used in the YSBL with the µplate (Korczyńska *et al.*, 2007 ▶), which consumes a maximum of 1.2 µl reservoir solution per condition. As a result, glucosamine-PGA-LM and glucosamine-PGA-HM were tested further, not as the main precipitants, but as high-molecular-weight additives to other PEGs (Table 4 ▶).

### 3.4. Formulation of the final PGA-based screen

As combination of glucosamine-PGA-LM/glucosamine-PGA-HM with PEGs was also highly effective in obtaining test crystals, the combination of nonmodified PGAs and PEG was also explored, resulting in the formulation of the ‘final’ PGA-based (PGA screen) crystallization matrix (Table 5 ▶). For practical reasons (*e.g.* high viscosity) and as a result of a very similar efficiency in crystallization properties, we decided to base the PGA screen entirely around PGA-LM rather than PGA-HM. This does not exclude PGA-HM (or glucosamine-PGAs) from future possible applications; instead, this decision acknowledges that PGA-LM can act very effectively for all PGA biopolymers in the initial crystallization screening. The final PGA screen combines several features of the PGA polymers that have been identified here as being useful for protein crystallization. These include (i) the capability of PGA to work as a stand-alone new protein precipitant (columns 1–2 in Table 5 ▶), (ii) its easy mixing properties with other PEGs (columns 3–4) and (iii) its stability in the presence of salts and other additives (columns 5–12). It is likely that the efficiency of the combination of PGA and PEGs derives from the co-existence of these two very different (especially in terms of molecular size) polymer-based crystallization media instead of their cumulative/additive effect. The large physico-chemical differences between these two polymers, in combination with their similar effectiveness in inducing protein crystallization, make them attractive candidates for further experimentations with screens (*e.g.* different molar ratios, different additives, salts *etc*.). Surprisingly, the crystallization efficiency, understood here simply as the minimum polymer concentration yielding crystals, was not very narrow in case of PGA-LM (or any other of the PGAs); crystals of the same test protein were obtained over a wide PGA concentration [*e.g.* ∼1–20%(*w*/*v*)]. Therefore, PGA-LM is used in columns 1–6 of the PGA screen at two very different concentrations to maximize the chance of nucleation and crystal growth.

### 3.5. Implications of PGAs for protein crystallization

We want to stress here that the ultimate and most stringent assessment of PGA in protein crystallization (for example *via* PGA-containing screens) can only be carried out on previously uncrystallized proteins. As previously stated, the test proteins employed here have mostly served as a preliminary assessment of the crystallization capabilities of the PGAs rather than as the most reliable means for the formulation of the PGA screen. As most of the YSBL-based research is focused on well defined targets, a genomics-type range of new proteins was not available here for extensive real-life scenario tests. Nonetheless, a PGA screen (as well as other PGAs screens described here) was tested on ten previously uncrystallized proteins from the YSBL including two bona fide membrane proteins. The results with the membrane proteins were especially encouraging as ∼25% of the conditions of the PGA screen yielded diffraction-quality crystals (the crystallization details and structure solutions will be described elsewhere). Consequently, we believe that the PGA test data presented here strongly indicate the potential usefulness of PGA in protein crystallization.

It is currently difficult to assess the mechanism by which PGAs induce protein crystallization. Their overall similarity to PEGs (linearity, hydrophilic/solubility profiles) may suggest the volume-exclusion mechanism proposed for PEGs (McPherson, 1998 ▶). However, it can be also envisaged that the polypeptide-like PGAs may have a much more dramatic impact on the rearrangement of structured water shells around proteins, a process that has been indicated as crucial for favourable thermodynamics of protein crystallization (Vekilov, 2007 ▶; Derewenda & Vekilov, 2006 ▶).

The properties of the PGAs and their easy accessibility make them, in our opinion, exciting and new crystallization polymers. We therefore would like to bring them to the attention of the crystallization community at this initial stage of exploration in order to encourage their future validation and trials in other laboratories.

All PGAs described here were tested for cryoprotection capabilities at a wide range of concentrations, but they did not radically help crystal vitrification. They are not obvious cryoprotectants as are 200–6K molecular-weight PEGs at higher ∼25–40%(*w*/*v*) concentrations. However, this part of the research is still under exploration.

In summary, we report here the development and testing of a new type of macromolecular precipitant based on poly-γ-­glutamic acid. Despite large molecular weights ranging from ∼200 kDa to over 1000 kDa and intrinsic heterogeneity of the molecular-weight distribution, these PGAs showed very encouraging effectiveness in the crystallization process as additives or stand-alone precipitants. The screens presented here, including the ‘final’ PGA screen, represent only preliminary exploration of PGAs rather than ultimate PGA-based crystallization protocols. This very application-driven report highlights the need for further investigations on the effects of PGAs on protein structure and function. It is possible that they have other applications as protein stabilisers, folding facilitators (macromolecular chaperone effects), simulators of protein cellular matrix *etc*. There is still great and unexplored potential in crystallization chemistry that merits urgent attention to facilitate the further progress of structural biology.

## Figures and Tables

**Table 1:** The template PGA screen

	1	2	3	4	5	6
	5%(*w*/*v*) PGA-LM			5%(*w*/*v*) PGA-HM		
	0.1 *M* NaOAc pH 4.5	0.1 *M* MES pH 6.5	0.1 *M* Tris pH 7.8	0.1 *M* NaOAc pH 4.5	0.1 *M* MES pH 6.5	0.1 *M* Tris pH 7.8
*A*	0.2 *M* MgCl_2_					
*B*	0.2 *M* (NH_4_)_2_SO_4_					
*C*	0.2 *M* Li_2_SO_4_					
*D*	0.6 *M* Na_2_SO_4_					
*E*	0.6 *M* sodium formate					
*F*	0.2 *M* zinc acetate					
*G*	0.2 *M* KSCN					
*H*	0.2 *M* KBr					

**Table 2:** The 96-condition carbohydrate–PGA screen

	1	2	3	4	5	6	7	8	9	10	11	12
	5%(*w*/*v*) PGA-LM			5%(*w*/*v*) PGA-HM			5%(*w*/*v*) PGA-LM			5%(*w*/*v*) PGA-HM		
	0.1 *M* NaOAc pH 4.5	0.1 *M* MES pH 6.5	0.1 *M* Tris pH 7.8	0.1 *M* NaOAc pH 4.5	0.1 *M* MES pH 6.5	0.1 *M* Tris pH 7.8	0.1 *M* NaOAc pH 4.5	0.1 *M* MES pH 6.5	0.1 *M* Tris pH 7.8	0.1 *M* NaOAc pH 4.5	0.1 *M* MES pH 6.5	0.1 *M* Tris pH 7.8
*A*	0.2 *M* MgCl_2_ + 1%(*w*/*v*) pectin						0.2 *M* MgCl_2_ + 5%(*w*/*v*) gum arabic					
*B*	0.2 *M* (NH_4_)_2_SO_4_ + 1%(*w*/*v*) pectin						0.2 *M* (NH_4_)_2_SO_4_ + 5%(*w*/*v*) gum arabic					
*C*	0.2 *M* Li_2_SO_4_ + 1%(*w*/*v*) pectin						0.2 *M* Li_2_SO_4_ + 5%(*w*/*v*) gum arabic					
*D*	0.6 *M* Na_2_SO_4_ + 1%(*w*/*v*) pectin						0.6 *M* Na_2_SO_4_ + 5%(*w*/*v*) gum arabic					
*E*	0.6 *M* sodium formate + 1%(*w*/*v*) pectin						0.6 *M* sodium formate + 5%(*w*/*v*) gum arabic					
*F*	0.2 *M* zinc acetate + 1%(*w*/*v*) pectin						0.2 *M* zinc acetate + 5%(*w*/*v*) gum arabic					
*G*	0.2 *M* KSCN + 1%(*w*/*v*) pectin						0.2 *M* KSCN + 5%(*w*/*v*) gum arabic					
*H*	0.2 *M* KBr + 1%(*w*/*v*) pectin						0.2 *M* KBr + 5%(*w*/*v*) gum arabic					

**Table 3:** The 16 glucosamine-PGA conditions Columns 1 and 2 contain the same salts at identical concentrations. Rows *E–H* in columns 1 and 2 also contain 10%(*w*/*v*) PEG 2K MME.

	1	2
	15%(*w*/*v*) glucosamine-PGA-LM	15%(*w*/*v*) glucosamine-PGA-HM
*A*	0.2 *M* (NH_4_)_2_SO_4_	
*B*	0.2 *M* sodium formate	
*C*	0.2 *M* MgCl_2_	
*D*	0.2 *M* KBr	
*E*	0.2 *M* (NH_4_)_2_SO_4_, 10%(*w*/*v*) PEG 2K MME	
*F*	0.2 *M* sodium formate, 10%(*w*/*v*) PEG 2K MME	
*G*	0.2 *M* MgCl_2_, 10%(*w*/*v*) PEG 2K MME	
*H*	0.2 *M* KBr, 10%(*w*/*v*) PEG 2K MME	

**Table 4:** 16 conditions that were found useful in combination of glucosamine-PGA with regular PEGs PEG 3350 was at 25%(*w*/*v*) and PEG 400 at 35%(*w*/*v*).

	1	2
	5%(*w*/*v*) glucosamine-PGA-LM	5%(*w*/*v*) glucosamine-PGA-HM
*A*	PEG 3350	PEG 3350
*B*	PEG 400	PEG 400
*C*	PEG 3350, 0.2 *M* MgCl_2_	PEG 3350, 0.2 *M* MgCl_2_
*D*	PEG 400, 0.2 *M* MgCl_2_	PEG 400, 0.2 *M* MgCl_2_
*E*	PEG 3350, 10% Tacsimate	PEG 3350, 10% Tacsimate
*F*	PEG 400, 10% Tacsimate	PEG 400, 10% Tacsimate
*G*	PEG 3350, 0.2 *M* KSCN	PEG 3350, 0.2 *M* KSCN
*H*	PEG 400, 0.2 *M* KSCN	PEG 400, 0.2 *M* KSCN

**Table:** All PEG concentrations are given in %(*w*/*v*); glycerol, MPD and 1,2,6-hexanetriol (HXT) concentrations are given in %(*v*/*v*).

	1	2	3	4	5	6
	8% PGA-LM	16% PGA-LM	5% PGA-LM	15% PGA-LM	5% PGA-LM	15% PGA-LM
*A*	0.3 *M* KBr	0.3 *M* KBr	20% PEG 1K	20% PEG 1K	30% PEG 200	20% PEG 200
*B*	0.2 *M* MgCl_2_	0.2 *M* MgCl_2_	20% PEG 2K MME	20% PEG 2K MME	30% PEG 400	20% PEG 400
*C*	10% Tacsimate	10% Tacsimate	20% PEG 3350	20% PEG 3350	30% PEG 550 MME	20% PEG 550 MME
*D*	0.2 *M* Na formate	0.2 *M* Na formate	15% PEG 4K	15% PEG 4K	30% PEG 750 MME	20% PEG 750 MME
*E*	0.4 *M* NH_4_ formate	0.4 *M* NH_4_ formate	20% PEG 5K MME	20% PEG 5K MME	30% PEG 600	20% PEG 600
*F*	0.2 *M* KSCN	0.2 *M* KSCN	15% PEG 6K	15% PEG 6K	30% MPD	20% MPD
*G*	0.2 *M* proline	0.2 *M* proline	12% PEG 8K	12% PEG 8K	20% HXT	20% HXT
*H*	0.2 *M* arginine	0.2 *M* arginine	10% PEG 20K	10% PEG 20K	20% glycerol	20% glycerol

**Table:** 

	7	8	9	10	11	12
	10% PGA-LM, 10% Tacsimate	10% PGA-LM, 10% Tacsimate	10% PGA-LM, 0.3 *M* KBr	10% PGA-LM, 0.3 *M* KBr	10% PGA-LM, 0.2 *M* KSCN	10% PGA-LM, 0.2 *M* KSCN
*A*	20% PEG 1K	30% PEG 200	20% PEG 1K	30% PEG 200	20% PEG 1K	30% PEG 200
*B*	20% PEG 2K MME	30% PEG 400	20% PEG 2K MME	30% PEG 400	20% PEG 2K MME	30% PEG 400
*C*	20% PEG 3350	30% PEG 550 MME	20% PEG 3350	30% PEG 550 MME	20% PEG 3350	30% PEG 550 MME
*D*	15% PEG 4K	30% PEG 750 MME	15% PEG 4K	30% PEG 750 MME	15% PEG 4K	30% PEG 750 MME
*E*	20% PEG 5K MME	30% PEG 600	20% PEG 5K MME	30% PEG 600	20% PEG 5K MME	30% PEG 600
*F*	15% PEG 6K	30% MPD	15% PEG 6K	30% MPD	15% PEG 6K	30% MPD
*G*	12% PEG 8K	20% HXT	12% PEG 8K	20% HXT	12% PEG 8K	20% HXT
*H*	10% PEG 20K	20% glycerol	10% PEG 20K	20% glycerol	10% PEG 20K	20% glycerol
